# Role of Biomarkers in Prediction of Cardiotoxicity During Cancer Treatment

**DOI:** 10.1007/s11936-018-0641-z

**Published:** 2018-06-19

**Authors:** Li-Ling Tan, Alexander R. Lyon

**Affiliations:** 1 Cardio-Oncology Service, Department of Cardiology, Royal Brompton Hospital, London, SW3 6NP UK; 2 Department of Cardiology, National University Heart Centre Singapore, Singapore, Singapore; 3 National Heart and Lung Institute, Imperial College London, London, UK

**Keywords:** Biomarkers, Troponin, Brain natriuretic peptide, Chemotherapy, Cardiotoxicity

## Abstract

**Purpose of review:**

As cancer survivor rates improve with early screening and modern treatment options, cardiotoxicity is becoming an increasing problem. It is imperative for physicians to recognize adverse events early so that appropriate measures can be taken before advanced and permanent cardiac dysfunction ensues. In this review, we will evaluate the literature surrounding current cardiac biomarkers in the detection of cardiotoxicity during cancer treatment as well as discuss the role of emerging novel biomarkers.

**Recent findings:**

Troponin and brain natriuretic peptides show promise in the detection of subclinical cardiotoxicity during cancer treatment. In addition to identifying late complications among cancer survivors, they have the potential to predict patients who are at risk of developing cardiotoxicity prior to the initiation of cancer therapy. However, there are also conflicting data due to varying study design.

**Summary:**

Although biomarkers are an attractive option in the detection of cardiotoxicity among cancer patients, current recommendations surrounding its role are based on expert consensus opinion. Further research with appropriately designed prospective trials is required to guide optimal clinical practice.

## Introduction

Conventional chemotherapy, radiotherapy, and surgery were previously the mainstay treatment options available to cancer patients. However, rapid advancements in medical science have led to the development of novel molecular targeted therapies that specifically focus on the biological pathways of cancer cells while sparing the healthy cells. However, due to complex and tightly intertwined cellular regulation processes between cancer cell proliferation and cardiovascular biology, many have resulted in cardiotoxicity.

Cancer drug-induced cardiotoxicity (CDIC) and cancer radiation-induced cardiotoxicity (CRIC) are burgeoning clinical problems. Heart failure is the most common form of CDIC, but this field also comprises hypertension, arrhythmias, ischemia, thromboembolism, valvular heart disease, and sudden cardiac death. Some of these cardiac complications such as arrhythmias and ischemia can occur acutely during cancer treatment, but others such as heart failure and CRIC usually present years after completion of treatment. These cardiac sequelae are more apparent now because early malignancy detection and more efficacious cancer therapies have substantially improved cancer survivorship.

In the United Kingdom, the percentage of breast cancer patients who live at least 10 years beyond their diagnosis date has risen from 40% in the early 1970s to 78% in 2010 [1], and the 5-year survival rate for childhood cancers diagnosed from 2006 to 2010 has reached 82% [2]. In addition, co-morbidities that occur with aging (e.g., hypertension, diabetes, coronary artery disease) further increase the risk of CDIC and CRIC among cancer survivors [3–5]. Cardiovascular disease is the leading cause of non-cancer-related mortality in breast cancer survivors, making up 35% of deaths in those aged 50 years and above [6]. The risk of cardiovascular-related death is higher in breast cancer survivors compared to women without breast cancer, and this increased risk typically occurs 7 years after the diagnosis of cancer [7].

## Current landscape

Anthracycline cardiomyopathy is the CDIC which has been most extensively studied. Historically, anthracycline cardiomyopathy was regarded as late in onset, progressive, and irreversible in nature [8]. Unfortunately, unlike ischemic or idiopathic dilated cardiomyopathy, heart failure therapies were less effective in improving the prognosis of patients with advanced anthracycline cardiomyopathy presenting late after cancer treatment [9]. Modern and contemporary approaches now allow detection of milder forms at an earlier stage when recovery is possible, and permanent dysfunction can be avoided. As prevention rather than rescue therapy is now the preferred strategy, physicians need tools to accurately predict patients with the highest risk of cardiotoxicity before the start of cancer treatment and also identify patients who develop subclinical cardiotoxicity early during the course of treatment. This allows for adjustment of chemotherapy regimens and/or initiation of cardioprotective strategies to reduce treatment-related mortality and morbidity without jeopardizing cancer prognosis.

There are presently varying definitions for CDIC. Typical histopathology features of anthracycline cardiomyopathy include loss of myofibrils and vacuolization of the cytoplasm [10], but endomyocardial biopsy is seldom utilized now due to its invasive nature. The Cardiac Review and Evaluation Committee supervising trastuzumab clinical trials defines left ventricular (LV) dysfunction according to clinical presentation and reduction in left ventricular ejection fraction (LVEF) [11]. On the other hand, in addition to clinical signs and symptoms, the latest Common Terminology Criteria for Adverse Events version 4 takes into account biomarker and cardiac imaging abnormalities when grading the severity of heart failure [12].

The American Society of Echocardiography and European Association of Cardiovascular Imaging define cancer therapeutics-related cardiac dysfunction as a > 10% drop in LVEF to below 53% [13]. However practically, an LVEF of 50–54% may be considered ‘low normal’ in many cardiology departments, and more sensitive parameters, e.g., global longitudinal strain, can clarify if function is normal or reduced in patients where their LVEF has fallen into the ‘low normal’ range. Below 50% is accepted as abnormal. Unfortunately, LVEF as a diagnostic tool for early cardiomyopathy has poor sensitivity and variable reproducibility. LVEF may still remain normal despite the presence of myocardial injury [14] because the heart undergoes ventricular remodeling in response to a cardiac insult in order to maintain adequate cardiac output [15]. A fall in LVEF is only appreciable on imaging modalities after substantial cardiac injury or dysfunction is established and compensatory mechanisms are exhausted. However, advanced cardiac dysfunction, particularly when presenting with clinically decompensated heart failure, becomes more refractory to cardiac treatment.

Biomarkers offer a promising alternative solution for the early detection of cardiotoxicity as they overcome the shortcomings associated with imaging. As with echocardiography and magnetic resonance imaging, there is no radiation exposure with biomarkers. However, biomarkers are easier to perform and less time consuming for patients as it only involves a blood draw that can ideally be timed with other blood tests requested by the physicians. Biomarkers are also more reproducible than imaging since it avoids the issues of load dependency, technical factors, and inter- and intra-operator variability during result interpretation. Therefore, there is growing interest in biomarkers as a tool for cardiotoxicity risk prediction before cancer therapy, early detection of subclinical events during treatment, and identification of late complications during cancer survivorship.

## Which cardiac biomarkers are currently helpful?

Biomarkers are measurable biologic variables that provide objective information about normal and pathogenic biological processes [16], and they now routinely refer to circulating serum and plasma biochemicals present within the body. The perfect biomarker should improve clinical outcomes through better risk stratification, diagnostic certainty, and monitoring of disease progression and treatment response beyond pre-existing tests [17]. The biomarker assay should also generate precise and reproducible results with a rapid turnaround time at an affordable cost [17].

Troponin and brain natriuretic peptides are the two most established cardiac biomarkers for acute coronary syndrome and heart failure, respectively. Currently, there are no fixed recommendations regarding biomarkers in the detection of CDIC and CRIC. Results from trials performed to date are inconsistent and lack comparability mainly due to the lack of standardization in trial methodology. There is wide heterogeneity in terms of malignancy types, cancer treatment schedules, and definition of cardiotoxicity across the trials. Each trial also adopts different biomarker assays with variable threshold values, and the biomarkers are also sampled at different frequencies and time points with respect to the treatment regimen. In this paper, we will review the evidence surrounding troponin and brain natriuretic peptides in the context of CDIC and CRIC and discuss the role of upcoming biomarkers (Table 1).

**Table 1:** Circulating biomarkers for cancer treatment-related cardiotoxicity

Troponin	
Conventional troponin I and T	
High-sensitive troponin I and T	
Natriuretic peptides	
BNP	
NT-proBNP	
Biomarkers under investigation	
Myeloperoxidase	
MicroRNA	
Peripheral blood mononuclear cell gene expression profile	
ST-2	
Galactin-3	
High-sensitive CRP	
GDF-15	

*BNP* brain natriuretic peptide, *CRP* C-reactive protein, *GDF-15* growth differentiation factor 15, *NT-proBNP* N-terminal pro-brain natriuretic peptide

## Troponin

Cardiac troponin, specifically troponin I (TnI) and troponin T (TnT), is found within cardiac myocytes and is released into the serum when there is a disruption in sacrolemmal integrity. Previously, only conventional troponin assays were available to detect cardiac troponin at serum concentrations of 100 ng/L or above. However, technological advancements have led to the development of newer generation assays known as high-sensitive troponin assays, which can detect troponin at much lower concentrations. In clinical practice, troponin is a sensitive and specific marker of myocardial injury and is routinely measured in acute myocardial infarction for diagnostic and prognostic purposes [18]. However, troponin can be elevated in other scenarios such as hypertensive emergencies, renal failure, rhabdomyolysis, sepsis, chronic poor vascular health, and drug-induced cardiotoxicity [19], hence accounting for the low specificity of high-sensitive troponin assays [20].

The utility of troponin in CDIC has been demonstrated in animal models. Herman et al. showed a positive correlation between rising TnI levels and cumulative doxorubicin doses in spontaneously hypertensive rats [21]. A significant increase in TnT level, in association with reduced left ventricular contractility, was observed in daunorubicin-treated rabbits as compared to controls [22]. Unfortunately, presence of troponin elevation implies that myocardial cell death has already occurred, hence raising questions about its role as an effective early marker of cardiotoxicity.

### Role of conventional troponin in anthracycline-induced cardiotoxicity

#### Troponin elevation during anthracycline treatment

Majority of biomarker studies are conducted in the setting of anthracycline exposure among cancer patients, and they generally show that early troponin elevation precedes changes in LVEF. One study looked at serial changes in TnI and LVEF after every cycle of high-dose anthracycline-containing chemotherapy in 204 cancer patients [23]. TnI was increased (> 0.4 ng/ml) in 32% of the patients. After 7 months following the completion of treatment, patients with elevated TnI had significantly greater LVEF reduction compared with those without TnI elevation. In another 703 patients with predominantly breast cancer and lymphoma, persistent TnI elevation (> 0.08 ng/ml) measured within 72 h of chemotherapy administration and at 1 month after the end of treatment portended the highest risk of cardiotoxicity (84%) over a mean follow-up duration of 20 months (*p* < 0.001) compared with patients with transient or no TnI elevation [24]. Cardiotoxicity in this study was defined as cardiac death, acute pulmonary edema, overt heart failure, asymptomatic LVEF reduction ≥ 25%, or life-threatening arrhythmias. TnT elevation was also observed in adult patients with hematological malignancies after a median of two anthracycline cycles, and a significantly greater reduction in LVEF on follow-up echocardiogram was seen in the patients with positive TnT compared to those without TnT rise (median LVEF reduction 10 versus 2%, *p* = 0.017) [25]. In addition, greater extent of troponin elevation reflects worse LV dysfunction [23, 26].

#### Biomarker-guided treatment strategies

The notion of initiating all cancer patients on cardioprotective strategies prior to cancer treatment is gaining interest. Lipshultz et al. showed that administrating dexrazoxane to children suffering from acute leukemia prior to doxorubicin chemotherapy resulted in a significantly lower incidence of elevated TnT compared to those who did not receive dexrazoxane [27]. The OVERCOME trial showed that pre-treating patients on high-dose chemotherapy with enalapril and carvedilol prevented deterioration in LVEF and led to statistically fewer deaths and heart failure events (*p* = 0.036) when compared to the control group [28]. However, there are concerns of subjecting low-risk patients to unnecessary adverse events such as hypotension and renal dysfunction, which are common with heart failure therapies. Further, hemodynamic compromise may also occur since cancer patients are prone to sepsis and dehydration.

The following study by Cardinale et al. illustrates how troponin can be used to select high-risk cancer patients and help tailor patient-specific treatments. One hundred and fourteen high-risk cancer patients identified based on having early TnI elevation soon after high-dose chemotherapy were randomized to receive either 1-year treatment of enalapril or no treatment (control group) [29]. At the end of 1 year, the enalapril group had significantly less cardiotoxicity than the control group (0 versus 43%; *p* < 0.001). LVEF and LV volumes remained preserved in the enalapril group while the control group had progressive LVEF reduction and increase in LV volumes compared to baseline. The enalapril group also had significantly less heart failure and arrhythmic events (*p* < 0.001 and *p* = 0.01, respectively) than the control group. Conversely, negative troponin values are also of equal importance—its high negative predictive value implies that such patients are at very low risk of cardiotoxicity [24], and therefore are unlikely to require vigilant surveillance, cardioprotective medications, or cessation of their potentially curable cancer drug.

### Role of conventional troponin in cardiotoxicity associated with other cancer therapies

Evidence for the utility of troponin in detecting early cardiotoxicity with newer targeted cancer therapies is less robust compared with anthracyclines. Apart from predicting an increased risk of cardiotoxicity in breast cancer patients receiving trastuzumab (hazard ratio [HR] 22.9; 95% confidence interval [CI], 11.6 to 45.5; *p* < 0.001), TnI elevation was also associated with a reduced likelihood of LVEF recovery (HR 2.88; 95% CI, 1.78 to 4.63; *p* < 0.001) [30]. However, in this study, 19% of TnI elevations occurred before trastuzumab treatment, suggesting that subclinical cardiac dysfunction due to other reasons (e.g., previous anthracycline, non-cardiac illness) was already present at baseline. Among 90 patients receiving molecular targeted therapies for metastatic solid tumors in a phase I trial, TnI elevation occurred in 11%, but this was not associated with clinically apparent cardiac events [31]. In another observational single-center study, 9 out of 86 metastatic renal cell carcinoma patients on sorafenib or sunitinib experienced a rise in TnT [32]. However, the magnitude of troponin increase was minimal, the sample population was small, and it was not clear if this correlated with LVEF reduction.

There is some data regarding the role of troponin in the detection of CRIC. Two studies looking at the effects of radiotherapy in breast cancer patients found that increased troponin levels occurred with higher cardiac radiation doses and it was also associated with deterioration in echocardiographic strain and diastology parameters [33, 34]. Unfortunately, the study populations were small, and the follow-up duration was too short to assess long-term clinical outcomes.

### Role of conventional troponin in cancer survivorship

Expert groups acknowledge the need for surveillance for asymptomatic late cardiotoxicity in cancer survivors [35–37], especially in the pediatric cohort where close to 60% have been exposed to anthracycline and/or cardiac irradiation [38]. The experts agree that cardiac biomarkers should be performed. However, there are no formal guidelines advising on which biomarker to monitor and the surveillance frequency as supporting evidence is modest. Several studies have demonstrated the lack of correlation between troponin and LV dysfunction in childhood cancer survivors [39–41].

## High-sensitive troponin

There are several reasons why some trials fail to demonstrate an association between conventional troponin and LV dysfunction [42–45]. As previously mentioned, one explanation is the use of different biomarker assays, specifically the lower sensitivity conventional troponin assays. Troponin elevation in CDIC is often mild [46] and may not be detectable on conventional assays. However, the newer high-sensitive troponin assays can detect troponin at very low concentrations [20]. With increased sensitivity comes reduced specificity, and unfortunately not every raised reading using high-sensitive troponin assays in cancer patients reflects CDIC. Concomitant medical conditions such as acute kidney injury, sepsis, pulmonary embolism, and tachyarrhythmias are common in cancer patients and can increase troponin levels. In addition, there is the issue of biological variance in healthy individuals [47]. Hence, physicians need to interpret positive troponin results with care and in the appropriate clinical context.

The use of high-sensitive troponin assays has since been integrated into several studies. Sawaya and his colleagues found that elevated high-sensitive troponin levels, together with echocardiographic markers of myocardial deformation, predicted the occurrence of cardiotoxicity among breast cancer patients receiving anthracycline and trastuzumab [48, 49], while Ky et al. demonstrated that an early rise in high-sensitivity TnI from baseline to 3 months was associated with an increased cardiotoxicity risk among similar patients [50•]. In a subgroup analysis of the herceptin adjuvant (HERA) study, the presence of raised high-sensitive troponin before trastuzumab therapy was associated with a 2.4- to 4.5-fold increased risk of subsequent LVEF deterioration [51].

## Natriuretic peptides

Brain natriuretic peptide (BNP), together with its inactive N-terminal amino acid fragment NT-proBNP, is released by the ventricles in response to volume overload and/or wall stress [52]. BNP induces natriuresis and diuresis in order to maintain euvolemia. Current guidelines recommend measuring natriuretic peptide levels in patients with heart failure as it provides valuable diagnostic, therapeutic, and prognostic information [53, 54].

### Role of natriuretic peptides in anthracycline-induced cardiotoxicity

Natriuretic peptides are the next most commonly researched biomarker in the context of CRIC apart from troponin. They have been shown to be more sensitive markers of cardiotoxicity than echocardiography in several studies. In a prospective study by De Iuliis et al., NT-proBNP was significantly elevated at multiple time points after the completion of chemotherapy whereas there was no significant change in LVEF [55]. The authors also showed that NT-proBNP was predictive of mortality at 1 year [55]. Another study showed that persistent elevation of NT-proBNP taken early after high-dose chemotherapy was significantly associated with the development of LV systolic and diastolic dysfunction at 1-year follow-up [56]. Among 205 children receiving doxorubicin for acute leukemia, elevation of NT-proBNP within the first 90 days of chemotherapy was predictive of cardiac dysfunction on echocardiography 4 years later [57]. A recent study investigated whether serial BNP measurements taken before and after every cycle of anthracycline throughout the entire course of therapy predicted cardiotoxicity in 109 patients with primarily sarcoma and lymphoma [58]. Results showed that in comparison to patients without cardiac events, BNP levels were significantly higher before and after every cycle of anthracycline in patients who experienced cardiac events (defined as asymptomatic LV dysfunction, symptomatic heart failure, symptomatic arrhythmia, acute coronary syndrome, or sudden cardiac death). There was also a tendency for patients with cardiac events to have a lower LVEF at the end of follow-up (*p* = 0.05) [58].

### Role of natriuretic peptides in cardiotoxicity associated with other cancer therapies

In a single-center study consisting of 159 metastatic renal cell carcinoma patients treated with tyrosine kinase inhibitors and other targeted therapies, 43 patients (27%) developed asymptomatic cardiotoxicity defined by elevated NT-proBNP level and/or LVEF impairment [59]. Twelve out of the 38 patients who had increased NT-proBNP levels also developed reduction in LVEF [59]. In another group of patients receiving sunitinib for metastatic renal cell carcinoma, the risk of LV dysfunction was highest within the first treatment cycle [60]. Although there was a statistically significant LVEF drop of 1.9% from baseline after the first treatment cycle (95% CI, − 3.2 to − 0.5, *p* = 0.007), this reduction was clinically not meaningful and was only transient after appropriate cardiac therapies. The mean BNP level did not show any significant change from baseline during the course of sunitinib treatment [60].

Raised natriuretic peptide levels have also been reported in patients treated with thoracic irradiation and may reflect early cardiotoxicity before LVEF [61]. Patients who received radiotherapy for left-sided breast cancer had significantly higher NT-proBNP levels at 6 months post-irradiation than those without radiotherapy [62]. Increased doses of cardiac irradiation were also associated with a greater magnitude of natriuretic peptide elevation [62, 63].

### Role of natriuretic peptides in cancer survivorship

As discussed earlier, the utility of troponin in long-term surveillance of cancer survivors seems weak. However, brain natriuretic peptides appear to be potential early indicators of late cardiotoxicity during cancer survivorship as they have been associated with increased LV dimensions and echocardiographic features of systolic and diastolic dysfunction [39–41, 56, 57, 64, 65].

### Limitations of natriuretic peptides

There are investigators who fail to find an association between natriuretic peptides and cardiotoxicity [48, 49, 50•, 66]. Reasons for conflicting natriuretic peptide trial results are similar to the troponin trials: retrospective design, small sample size, and lack of standardized biomarker reference ranges. Elderly individuals and females also have higher normal natriuretic peptide levels [67, 68], and worsening renal function increases natriuretic peptide levels [69]. Recent evidence also suggests that cancer itself may potentially increase BNP levels through cancer-associated inflammation, with patients suffering from metastatic disease having significantly higher BNP levels than those without metastatic involvement [70]. These confounding factors should definitely be taken into consideration during the interpretation of results.

## New biomarkers in the pipeline

There are several attractive biomarkers that show promise as future predictors of CDIC.

### Myeloperoxidase

Myeloperoxidase (MPO) is regarded as a marker of oxidative stress. It is released by neutrophils during periods of inflammation and oxidative stress [50•], and the generation of excess free oxygen radicals is hypothesized to be pivotal in the pathogenesis of anthracycline cardiotoxicity [71]. Among 78 breast cancer patients receiving doxorubicin and trastuzumab, an early rise in MPO levels from baseline to 3 months was associated with a higher risk of a first cardiotoxic event during the 15-month period of treatment [50•]. In a later study involving the same patient cohort, the same authors showed that increases in MPO beyond 3 months of chemotherapy still remained predictive of increased cardiotoxicity risk over the course of treatment (HR 1.38; 95% CI, 1.10 to 1.71, *p* = 0.02) [72].

### MicroRNAs

MicroRNAs are small, non-coding RNA molecules that play an important role in the regulation of gene expression. They are involved in numerous cellular processes including those of the cardiovascular system [73]. Within the heart itself, there is differential expression of microRNAs [74], and some microRNAs have been linked to certain cardiovascular diseases. At present, miR-1, miR-133, miR-208, and miR-499 are cardiac miRNAs that are most extensively investigated [75]. MiR-208 and miR-499, which are specifically found in cardiac myocytes, are significantly elevated in animal and human myocardial infarction models [76] while miR-1 has been shown to provide therapeutic information in the setting of heart failure [77].

Several studies have demonstrated encouraging results for the utility of microRNAs as biomarkers for CRIC. Doxorubicin caused upregulation of miR-146a in neonatal cardiac rat models [78]. A recent study involving 33 children showed elevation of plasma miR-29b and miR-499 levels after anthracycline exposure, and the degree of elevation positively correlated with anthracycline dose and troponin rise [79]. In another cohort of breast cancer patients receiving doxorubicin, an increase in miR-1 was significantly associated with LVEF reduction (*p* < 0.001) and miR-1 was superior to TnI in predicting cardiotoxicity risk in the study [80]. On the other hand, results of a study looking at the role of miR-208a in the setting of doxorubicin cardiotoxicity were disappointing [81]. Despite this, investigators should continue to evaluate the role of microRNAs as they surpass existing biomarkers in several important aspects: (i) MicroRNAs are present in multiple body fluids [82], (ii) they remain stable under extreme temperatures and pH, (iii) they have long half-life, (iv) they can be measured quantitatively using different methods [83].

### Peripheral blood mononuclear cell gene expression profile

Oxidative stress affects the immune response system. Patients exposed to continuous doxorubicin infusions have increased DNA-base oxidation within their peripheral blood mononuclear cells (PMBC) compared to pre-treatment [84]. These changes within the PMBC result in detectable alterations in their gene expression profile [85]. Todorova et al. found a high similarity between the gene expression profiles of PMBC and cardiac tissue in rats with doxorubicin cardiotoxicity, and the common genes identified were involved in oxidative stress response pathways [86]. A subsequent study by the same group demonstrated a unique gene expression profile that was observed in breast cancer patients who developed doxorubicin cardiotoxicity but absent in those with preserved LVEF [87].

### Other novel biomarkers

There are other emerging biomarkers such as ST2, galactin-3, high-sensitivity C-reactive protein (hs-CRP), and growth differentiation factor 15 (GDF-15) but results are less encouraging. Several studies involving ST2 and galactin-3, which are markers of fibrosis, showed no significant association with cardiotoxicity [45, 49, 50•]. Raised hs-CRP levels ≥ 3 mg/L were found to be predictive of reduced LVEF in breast cancer patients treated with trastuzumab in one study [44], but there was no correlation between hs-CRP and cardiotoxicity risk in another study [50•]. However, GDF-15, which is an indicator of inflammation and oxidative stress, shows promise in the detection of late anthracycline cardiotoxicity in pediatric cancer survivors [43].

## Future directions and conclusion

Circulating biomarkers are attractive tools in the detection of CDIC but no single measure is perfect. There are discordant data surrounding the utility of biomarkers in the pre-, during-, and post-cancer treatment periods. A multimodality strategy that incorporates several biomarkers with cardiac imaging appears to provide incremental value in highlighting patients at high risk of cardiotoxicity [49, 50•]. Genomics is a blossoming field, and the identification of genetic variants that predispose or protect one from adverse cardiotoxic side effects is a step towards precision medicine. An ongoing study by Skitch et al. aims to develop a risk prediction model for pediatric cancer patients through the integration of clinical risk factors, imaging parameters, biomarkers, and genomic factors [88].

Before formal practice guidelines regarding the role of biomarkers can be implemented, there are many unanswered questions that need to be tackled. There is a need for new biomarkers that can detect myocardial injury earlier than troponin. Factors such as feasibility of repeated blood sampling and issue of cost-effectiveness must be considered. It is also crucial to establish evidence-based clinical strategies to prevent or treat cardiotoxicity while patients continue to receive effective life-saving cancer therapies. The Cardiac CARE trial (ISRCTN24439460) is an example of an ongoing biomarker-guided cardioprotection study that randomizes breast cancer patients with elevated troponin levels to receive either heart failure therapy or standard care prior to the initiation of chemotherapy [89]. In summary, well-designed prospective trials with good statistical power and long-term follow-up are imperative to address these outstanding issues and hopefully they will provide physicians with the answers needed to manage this group of challenging patients.

## References

[1] Cancer Research UK. Breast cancer survival statistics. 2014 [cited 2017 5 November ]. Available from: http://www.cancerresearchuk.org/health-professional/cancer-statistics/statistics-by-cancer-type/breast-cancer/survival#heading-Two.

[2] Cancer Research UK. Children’s cancer survival statistics. 2015 [cited 2017 5 November ]. Available from: http://www.cancerresearchuk.org/health-professional/cancer-statistics/childrens-cancers/survival#heading-Zero.

[3] Szmit S, Jurczak W, Zaucha JM, Drozd-Sokolowska J, Spychalowicz W, Joks M, Dlugosz-Danecka M, Torbicki A (2014). Pre-existing arterial hypertension as a risk factor for early left ventricular systolic dysfunction following (R)-CHOP chemotherapy in patients with lymphoma. J Am Soc Hypertens.

[4] Pinder MC, Duan Z, Goodwin JS, Hortobagyi GN, Giordano SH (2007). Congestive heart failure in older women treated with adjuvant anthracycline chemotherapy for breast cancer. J Clin Oncol.

[5] Wang L, Tan TC, Halpern EF, Neilan TG, Francis SA, Picard MH, Fei H, Hochberg EP, Abramson JS, Weyman AE, Kuter I, Scherrer-Crosbie M (2015). Major cardiac events and the value of echocardiographic evaluation in patients receiving anthracycline-based chemotherapy. Am J Cardiol.

[6] Schairer C, Mink PJ, Carroll L, Devesa SS (2004). Probabilities of death from breast cancer and other causes among female breast cancer patients. J Natl Cancer Inst.

[7] Bradshaw PT, Stevens J, Khankari N, Teitelbaum SL, Neugut AI, Gammon MD (2016). Cardiovascular disease mortality among breast Cancer survivors. Epidemiology.

[8] Yeh ET, Bickford CL (2009). Cardiovascular complications of cancer therapy: incidence, pathogenesis, diagnosis, and management. J Am Coll Cardiol.

[9] Chatterjee K, Zhang J, Honbo N, Karliner JS (2010). Doxorubicin cardiomyopathy. Cardiology.

[10] Singal PK, Iliskovic N (1998). Doxorubicin-induced cardiomyopathy. N Engl J Med.

[11] Seidman A, Hudis C, Pierri MK, Shak S, Paton V, Ashby M, Murphy M, Stewart SJ, Keefe D (2002). Cardiac dysfunction in the Trastuzumab clinical trials experience. J Clin Oncol.

[12] National Cancer Institute. Common Terminology Criteria for Adverse Events (CTCAE). 2009 [cited 2017 21 November]. Available from: https://evs.nci.nih.gov/ftp1/CTCAE/CTCAE_4.03_2010-06-14_QuickReference_5x7.pdf.

[13] Plana JC, Galderisi M, Barac A, Ewer MS, Ky B, Scherrer-Crosbie M, Ganame J, Sebag IA, Agler DA, Badano LP, Banchs J, Cardinale D, Carver J, Cerqueira M, JM DC, Edvardsen T, Flamm SD, Force T, Griffin BP, Jerusalem G, Liu JE, Magalhaes A, Marwick T, Sanchez LY, Sicari R, Villarraga HR, Lancellotti P (2014). Expert consensus for multimodality imaging evaluation of adult patients during and after cancer therapy: a report from the American Society of Echocardiography and the European Association of Cardiovascular Imaging. J Am Soc Echocardiogr.

[14] Ewer M, Ali MK, Mackay B, Wallace S, Valdivieso M, Legha SS, Benjamin RS, Haynie TP (1984). A comparison of cardiac biopsy grades and ejection fraction estimations in patients receiving Adriamycin. J Clin Oncol.

[15] Roca-Alonso L, Pellegrino L, Castellano L, Stebbing J (2012). Breast cancer treatment and adverse cardiac events: what are the molecular mechanisms?. Cardiology.

[16] Biomarkers Definitions Working G (2001). Biomarkers and surrogate endpoints: preferred definitions and conceptual framework. Clin Pharmacol Ther.

[17] Morrow DA, de Lemos JA (2007). Benchmarks for the assessment of novel cardiovascular biomarkers. Circulation.

[18] Roffi M, Patrono C, Collet JP, Mueller C, Valgimigli M, Andreotti F, Bax JJ, Borger MA, Brotons C, Chew DP, Gencer B, Hasenfuss G, Kjeldsen K, Lancellotti P, Landmesser U, Mehilli J, Mukherjee D, Storey RF, Windecker S, Baumgartner H, Gaemperli O, Achenbach S, Agewall S, Badimon L, Baigent C, Bueno H, Bugiardini R, Carerj S, Casselman F, Cuisset T, Erol C, Fitzsimons D, Halle M, Hamm C, Hildick-Smith D, Huber K, Iliodromitis E, James S, Lewis BS, Lip GY, Piepoli MF, Richter D, Rosemann T, Sechtem U, Steg PG, Vrints C, Luis Zamorano J, Management of Acute Coronary Syndromes in Patients Presenting without Persistent STSEotESoC (2016). 2015 ESC guidelines for the management of acute coronary syndromes in patients presenting without persistent ST-segment elevation: task force for the Management of Acute Coronary Syndromes in patients presenting without persistent ST-segment elevation of the European Society of Cardiology (ESC). Eur Heart J.

[19] Wallace KB, Hausner E, Herman E, Holt GD, MacGregor JT, Metz AL, Murphy E, Rosenblum IY, Sistare FD, York MJ (2004). Serum troponins as biomarkers of drug-induced cardiac toxicity. Toxicol Pathol.

[20] Marini MG, Cardillo MT, Caroli A, Sonnino C, Biasucci LM (2013). Increasing specificity of high-sensitivity troponin: new approaches and perspectives in the diagnosis of acute coronary syndromes. J Cardiol.

[21] Herman E, Lipshultz SE, Rifai N, Zhang J, Papoian T, Yu Z, Takeda K, Ferrams VJ (1998). Use of cardiac troponin T levels as an Indicatior of doxorubicin-induced cardiotoxicity. Cancer Res.

[22] Simunek T, Klimtova I, Adamcova M, Gersl V, Hrdina R, Sterba M, Kaplanova J, Mazurova Y (2003). Cardiac troponin T as an indicator of reduced left ventricular contractility in experimental anthracycline-induced cardiomyopathy. Cancer Chemother Pharmacol.

[23] Cardinale D, Sandri MT, Martinoni A, Tricca LabTech A, Civelli M, Lamantia G, Cinieri S, Martinelli G, Cipolla CM, Fiorentini C (2000). Left ventricular dysfunction predicted by early troponin I release after high-dose chemotherapy. J Am Coll Cardiol.

[24] Cardinale D, Sandri MT, Colombo A, Colombo N, Boeri M, Lamantia G, Civelli M, Peccatori F, Martinelli G, Fiorentini C, Cipolla CM (2004). Prognostic value of troponin I in cardiac risk stratification of cancer patients undergoing high-dose chemotherapy. Circulation.

[25] Auner HW, Tinchon C, Linkesch W, Tiran A, Quehenberger F, Link H, Sill H (2003). Prolonged monitoring of troponin T for the detection of anthracycline cardiotoxicity in adults with hematological malignancies. Ann Hematol.

[26] Garrone O, Crosetto N, Lo Nigro C, Catzeddu T, Vivenza D, Monteverde M, Merlano M, Feola M (2012). Prediction of anthracycline cardiotoxicity after chemotherapy by biomarkers kinetic analysis. Cardiovasc Toxicol.

[27] Lipshultz SE, Rifai N, Dalton VM, Levy DE, Silverman LB, Lipsitz SR, Colan SD, Asselin BL, Barr RD, Clavell L, Hurwitz CA, Moghrabi A, Samson Y, Schorin M, Gelber RD, Sallan SE (2004). The effect of Dexrazoxane on myocardial injury in doxorubicin-treated children with acute lymphoblastic Leukaemia. N Engl J Med.

[28] Bosch X, Rovira M, Sitges M, Domenech A, Ortiz-Perez JT, de Caralt TM, Morales-Ruiz M, Perea RJ, Monzo M, Esteve J (2013). Enalapril and carvedilol for preventing chemotherapy-induced left ventricular systolic dysfunction in patients with malignant hemopathies: the OVERCOME trial (preventiOn of left ventricular dysfunction with Enalapril and caRvedilol in patients submitted to intensive ChemOtherapy for the treatment of malignant hEmopathies). J Am Coll Cardiol.

[29] Cardinale D, Colombo A, Sandri MT, Lamantia G, Colombo N, Civelli M, Martinelli G, Veglia F, Fiorentini C, Cipolla CM (2006). Prevention of high-dose chemotherapy-induced cardiotoxicity in high-risk patients by angiotensin-converting enzyme inhibition. Circulation.

[30] Cardinale D, Colombo A, Torrisi R, Sandri MT, Civelli M, Salvatici M, Lamantia G, Colombo N, Cortinovis S, Dessanai MA, Nole F, Veglia F, Cipolla CM (2010). Trastuzumab-induced cardiotoxicity: clinical and prognostic implications of troponin I evaluation. J Clin Oncol.

[31] Ederhy S, Massard C, Dufaitre G, Balheda R, Meuleman C, Rocca CG, Izzedine H, Cohen A, Soria JC (2012). Frequency and management of troponin I elevation in patients treated with molecular targeted therapies in phase I trials. Investig New Drugs.

[32] Schmidinger M, Zielinski CC, Vogl UM, Bojic A, Bojic M, Schukro C, Ruhsam M, Hejna M, Schmidinger H (2008). Cardiac toxicity of sunitinib and sorafenib in patients with metastatic renal cell carcinoma. J Clin Oncol.

[33] Erven K, Florian A, Slagmolen P, Sweldens C, Jurcut R, Wildiers H, Voigt JU, Weltens C (2013). Subclinical cardiotoxicity detected by strain rate imaging up to 14 months after breast radiation therapy. Int J Radiat Oncol Biol Phys.

[34] Skytta T, Tuohinen S, Boman E, Virtanen V, Raatikainen P, Kellokumpu-Lehtinen PL (2015). Troponin T-release associates with cardiac radiation doses during adjuvant left-sided breast cancer radiotherapy. Radiat Oncol.

[35] Armenian SH, Lacchetti C, Barac A, Carver J, Constine LS, Denduluri N, Dent S, Douglas PS, Durand JB, Ewer M, Fabian C, Hudson M, Jessup M, Jones LW, Ky B, Mayer EL, Moslehi J, Oeffinger K, Ray K, Ruddy K, Lenihan D (2017). Prevention and monitoring of cardiac dysfunction in survivors of adult cancers: American Society of Clinical Oncology clinical practice guideline. J Clin Oncol.

[36] Curigliano G, Cardinale D, Suter T, Plataniotis G, de Azambuja E, Sandri MT, Criscitiello C, Goldhirsch A, Cipolla C, Roila F, Group EGW (2012). Cardiovascular toxicity induced by chemotherapy, targeted agents and radiotherapy: ESMO clinical practice guidelines. Ann Oncol.

[37] Virani SA, Dent S, Brezden-Masley C, Clarke B, Davis MK, Jassal DS, Johnson C, Lemieux J, Paterson I, Sebag IA, Simmons C, Sulpher J, Thain K, Thavendiranathan P, Wentzell JR, Wurtele N, Cote MA, Fine NM, Haddad H, Hayley BD, Hopkins S, Joy AA, Rayson D, Stadnick E, Straatman L (2016). Canadian cardiovascular society guidelines for evaluation and Management of Cardiovascular Complications of Cancer therapy. Can J Cardiol.

[38] Armenian SH, Hudson MM, Mulder RL, Chen MH, Constine LS, Dwyer M, Nathan PC, Tissing WJE, Shankar S, Sieswerda E, Skinner R, Steinberger J, van Dalen EC, van der Pal H, Wallace WH, Levitt G, Kremer LCM (2015). Recommendations for cardiomyopathy surveillance for survivors of childhood cancer: a report from the international late effects of childhood Cancer guideline harmonization group. Lancet Oncol.

[39] Armenian SH, Gelehrter SK, Vase T, Venkatramani R, Landier W, Wilson KD, Herrera C, Reichman L, Menteer JD, Mascarenhas L, Freyer DR, Venkataraman K, Bhatia S (2014). Screening for cardiac dysfunction in anthracycline-exposed childhood cancer survivors. Clin Cancer Res.

[40] Sherief LM, Kamal AG, Khalek EA, Kamal NM, Soliman AA, Esh AM (2012). Biomarkers and early detection of late onset anthracycline-induced cardiotoxicity in children. Hematology.

[41] Mavinkurve-Groothuis AM, Groot-Loonen J, Bellersen L, Pourier MS, Feuth T, Bokkerink JP, Hoogerbrugge PM, Kapusta L (2009). Abnormal NT-pro-BNP levels in asymptomatic long-term survivors of childhood cancer treated with anthracyclines. Pediatr Blood Cancer.

[42] Dodos F, Halbsguth T, Erdmann E, Hoppe UC (2008). Usefulness of myocardial performance index and biochemical markers for early detection of anthracycline-induced cardiotoxicity in adults. Clin Res Cardiol.

[43] Arslan D, Cihan T, Kose D, Vatansev H, Cimen D, Koksal Y, Oran B, Akyurek F (2013). Growth-differentiation factor-15 and tissue doppler imaging in detection of asymptomatic anthracycline cardiomyopathy in childhood cancer survivors. Clin Biochem.

[44] Onitilo AA, Engel JM, Stankowski RV, Liang H, Berg RL, Doi SAR (2012). High-sensitivity C-reactive protein (hs-CRP) as a biomarker for trastuzumab-induced cardiotoxicity in HER2-positive early-stage breast cancer: a pilot study. Breast Cancer Res Treat.

[45] van Boxtel W, Bulten BF, Mavinkurve-Groothuis AM, Bellersen L, Mandigers CM, Joosten LA, Kapusta L, de Geus-Oei LF, van Laarhoven HW (2015). New biomarkers for early detection of cardiotoxicity after treatment with docetaxel, doxorubicin and cyclophosphamide. Biomarkers.

[46] Christenson ES, James T, Agrawal V, Park BH (2015). Use of biomarkers for the assessment of chemotherapy-induced cardiac toxicity. Clin Biochem.

[47] Sherwood MW, Kristin NL (2014). High-sensitivity troponin assays: evidence, indications, and reasonable use. J Am Heart Assoc.

[48] Sawaya H, Sebag IA, Plana JC, Januzzi JL, Ky B, Cohen V, Gosavi S, Carver JR, Wiegers SE, Martin RP, Picard MH, Gerszten RE, Halpern EF, Passeri J, Kuter I, Scherrer-Crosbie M (2011). Early detection and prediction of cardiotoxicity in chemotherapy-treated patients. Am J Cardiol.

[49] Sawaya H, Sebag IA, Plana JC, Januzzi JL, Ky B, Tan TC, Cohen V, Banchs J, Carver JR, Wiegers SE, Martin RP, Picard MH, Gerszten RE, Halpern EF, Passeri J, Kuter I, Scherrer-Crosbie M (2012). Assessment of echocardiography and biomarkers for the extended prediction of cardiotoxicity in patients treated with anthracyclines, taxanes, and trastuzumab. Circ Cardiovasc Imaging.

[50] Ky B, Putt M, Sawaya H, French B, Januzzi JL, Sebag IA, Plana JC, Cohen V, Banchs J, Carver JR, Wiegers SE, Martin RP, Picard MH, Gerszten RE, Halpern EF, Passeri J, Kuter I, Scherrer-Crosbie M (2014). Early increases in multiple biomarkers predict subsequent cardiotoxicity in patients with breast cancer treated with doxorubicin, taxanes, and trastuzumab. J Am Coll Cardiol.

[51] Zardavas D, Suter TM, Van Veldhuisen DJ, Steinseifer J, Noe J, Lauer S, Al-Sakaff N, Piccart-Gebhart MJ, de Azambuja E (2017). Role of troponins I and T and N-terminal prohormone of brain natriuretic peptide in monitoring cardiac safety of patients with early-stage human epidermal growth factor receptor 2-positive breast Cancer receiving Trastuzumab: a Herceptin adjuvant study cardiac marker substudy. J Clin Oncol.

[52] Weber M, Hamm C (2006). Role of B-type natriuretic peptide (BNP) and NT-proBNP in clinical routine. Heart.

[53] Ponikowski P, Voors AA, Anker SD, Bueno H, Cleland JG, Coats AJ, Falk V, Gonzalez-Juanatey JR, Harjola VP, Jankowska EA, Jessup M, Linde C, Nihoyannopoulos P, Parissis JT, Pieske B, Riley JP, Rosano GM, Ruilope LM, Ruschitzka F, Rutten FH, van der Meer P, Authors/Task Force M. (2016). 2016 ESC guidelines for the diagnosis and treatment of acute and chronic heart failure: the task force for the diagnosis and treatment of acute and chronic heart failure of the European Society of Cardiology (ESC) developed with the special contribution of the heart failure association (HFA) of the ESC. Eur Heart J.

[54] Yancy CW, Jessup M, Bozkurt B, Butler J, Casey DE, Drazner MH, Fonarow GC, Geraci SA, Horwich T, Januzzi JL, Johnson MR, Kasper EK, Levy WC, Masoudi FA, McBride PE, McMurray JJ, Mitchell JE, Peterson PN, Riegel B, Sam F, Stevenson LW, Tang WH, Tsai EJ, Wilkoff BL, Writing Committee M, American College of Cardiology Foundation/American Heart Association Task Force on Practice G (2013). 2013 ACCF/AHA guideline for the management of heart failure: a report of the American College of Cardiology Foundation/American Heart Association task force on practice guidelines. Circulation.

[55] De Iuliis F, Salerno G, Taglieri L, De Biase L, Lanza R, Cardelli P, et al. Serum biomarkers evaluation to predict chemotherapy-induced cardiotoxicity in breast cancer patients. Tumour Biol. 2016 Mar;37(3):3379–87. 10.1007/s13277-015-4183-7 26449821

[56] Sandri MT, Salvatici M, Cardinale D, Zorzino L, Passerini R, Lentati P, Leon M, Civelli M, Martinelli G, Cipolla CM (2005). N-terminal pro-B-type natriuretic peptide after high-dose chemotherapy: a marker predictive of cardiac dysfunction?. Clin Chem.

[57] Lipshultz SE, Miller TL, Scully RE, Lipsitz SR, Rifai N, Silverman LB, Colan SD, Neuberg DS, Dahlberg SE, Henkel JM, Asselin BL, Athale UH, Clavell LA, Laverdiere C, Michon B, Schorin MA, Sallan SE (2012). Changes in cardiac biomarkers during doxorubicin treatment of pediatric patients with high-risk acute lymphoblastic leukemia: associations with long-term echocardiographic outcomes. J Clin Oncol.

[58] Lenihan DJ, Stevens PL, Massey M, Plana JC, Araujo DM, Fanale MA, Fayad LE, Fisch MJ, Yeh ET (2016). The utility of point-of-care biomarkers to detect cardiotoxicity during anthracycline chemotherapy: a feasibility study. J Card Fail.

[59] Hall PS, Harshman LC, Srinivas S, Witteles RM (2013). The frequency and severity of cardiovascular toxicity from targeted therapy in advanced renal cell carcinoma patients. JACC Heart Fail.

[60] Narayan V, Keefe S, Haas N, Wang L, Puzanov I, Putt M, Catino A, Fang J, Agarwal N, Hyman D, Smith AM, Finkelman BS, Narayan HK, Ewer S, ElAmm C, Lenihan D, Ky B (2017). Prospective evaluation of Sunitinib-induced cardiotoxicity in patients with metastatic renal cell carcinoma. Clin Cancer Res.

[61] Nellessen U, Zingel M, Hecker H, Bahnsen J, Borschke D (2010). Effects of radiation therapy on myocardial cell integrity and pump function: which role for cardiac biomarkers?. Chemotherapy.

[62] D'Errico MP, Grimaldi L, Petruzzelli MF, Gianicolo EA, Tramacere F, Monetti A, Placella R, Pili G, Andreassi MG, Sicari R, Picano E, Portaluri M (2012). N-terminal pro-B-type natriuretic peptide plasma levels as a potential biomarker for cardiac damage after radiotherapy in patients with left-sided breast cancer. Int J Radiat Oncol Biol Phys.

[63] D'Errico MP, Petruzzelli MF, Gianicolo EA, Grimaldi L, Loliva F, Tramacere F, Andreassi MG, Pili G, Picano E, Portaluri M (2015). Kinetics of B-type natriuretic peptide plasma levels in patients with left-sided breast cancer treated with radiation therapy: results after one-year follow-up. Int J Radiat Biol.

[64] Zidan A, Sherief LM, El-sheikh A, Saleh SH, Shahbah DA, Kamal NM, Sherbiny HS, Ahmad H (2015). NT-proBNP as early marker of subclinical late cardiotoxicity after doxorubicin therapy and mediastinal irradiation in childhood cancer survivors. Dis Markers.

[65] Yildirim A, Tunaoglu FS, Kanburoglu K, Pinarli FG (2013). The utility of NT-proBNP and various echocardiographic methods in the determination of doxorubicin induced subclinical late cardiotoxicity. Kardiol Pol.

[66] Ruggiero A, De Rosa G, Rizzo D, Leo A, Maurizi P, De Nisco A, Vendittelli F, Zuppi C, Mordente A, Riccardi R (2013). Myocardial performance index and biochemical markers for early detection of doxorubicin-induced cardiotoxicity in children with acute lymphoblastic leukaemia. Int J Clin Oncol.

[67] Cowie M (2003). Clinical applications of B-type natriuretic peptide (BNP) testing. Eur Heart J.

[68] Galasko GI, Lahiri A, Barnes SC, Collinson P, Senior R (2005). What is the normal range for N-terminal pro-brain natriuretic peptide? How well does this normal range screen for cardiovascular disease?. Eur Heart J.

[69] Takase H, Dohi Y (2014). Kidney function crucially affects B-type natriuretic peptide (BNP), N-terminal proBNP and their relationship. Eur J Clin Investig.

[70] Bando S, Soeki T, Matsuura T, Tobiume T, Ise T, Kusunose K, Yamaguchi K, Yagi S, Fukuda D, Iwase T, Yamada H, Wakatsuki T, Shimabukuro M, Muguruma N, Takayama T, Kishimoto I, Kangawa K, Sata M (2017). Plasma brain natriuretic peptide levels are elevated in patients with cancer. PLoS One.

[71] Vejpongsa P, Yeh ET (2014). Prevention of anthracycline-induced cardiotoxicity: challenges and opportunities. J Am Coll Cardiol.

[72] Putt M, Hahn VS, Januzzi JL, Sawaya H, Sebag IA, Plana JC, Picard MH, Carver JR, Halpern EF, Kuter I, Passeri J, Cohen V, Banchs J, Martin RP, Gerszten RE, Scherrer-Crosbie M, Ky B (2015). Longitudinal changes in multiple biomarkers are associated with cardiotoxicity in breast Cancer patients treated with doxorubicin, Taxanes, and Trastuzumab. Clin Chem.

[73] Small EM, Frost RJ, Olson EN (2010). MicroRNAs add a new dimension to cardiovascular disease. Circulation.

[74] Sandhu H, Maddock H (2014). Molecular basis of cancer-therapy-induced cardiotoxicity: introducing microRNA biomarkers for early assessment of subclinical myocardial injury. Clin Sci (Lond).

[75] Min PK, Chan SY (2015). The biology of circulating microRNAs in cardiovascular disease. Eur J Clin Investig.

[76] Wang GK, Zhu JQ, Zhang JT, Li Q, Li Y, He J, Qin YW, Jing Q (2010). Circulating microRNA: a novel potential biomarker for early diagnosis of acute myocardial infarction in humans. Eur Heart J.

[77] Vegter EL, van der Meer P, de Windt LJ, Pinto YM, Voors AA (2016). MicroRNAs in heart failure: from biomarker to target for therapy. Eur J Heart Fail.

[78] Horie T, Ono K, Nishi H, Nagao K, Kinoshita M, Watanabe S, Kuwabara Y, Nakashima Y, Takanabe-Mori R, Nishi E, Hasegawa K, Kita T, Kimura T (2010). Acute doxorubicin cardiotoxicity is associated with miR-146a-induced inhibition of the neuregulin-ErbB pathway. Cardiovasc Res.

[79] Leger KJ, Leonard D, Nielson D, de Lemos JA, Mammen PP, Winick NJ (2017). Circulating microRNAs: potential markers of cardiotoxicity in children and young adults treated with anthracycline chemotherapy. J Am Heart Assoc.

[80] Rigaud VO, Ferreira LR, Ayub-Ferreira SM, Avila MS, Brandao SM, Cruz FD, Santos MH, Cruz CB, Alves MS, Issa VS, Guimaraes GV, Cunha-Neto E, Bocchi EA (2017). Circulating miR-1 as a potential biomarker of doxorubicin-induced cardiotoxicity in breast cancer patients. Oncotarget.

[81] Oliveira-Carvalho V, Ferreira LR, Bocchi EA (2015). Circulating mir-208a fails as a biomarker of doxorubicin-induced cardiotoxicity in breast cancer patients. J Appl Toxicol.

[82] Weber JA, Baxter DH, Zhang S, Huang DY, Huang KH, Lee MJ, Galas DJ, Wang K (2010). The microRNA spectrum in 12 body fluids. Clin Chem.

[83] Ruggeri C, Gioffre S, Achilli F, Colombo GI, D'Alessandra Y. Role of microRNAs in doxorubicin-induced cardiotoxicity: an overview of preclinical models and cancer patients. Heart Fail Rev. 2017; 10.1007/s10741-017-9653-0 PMC5756562 28944400

[84] Doroshow JH, Synold TW, Somlo G, Akman SA, Gajewski E (2001). Oxidative DNA base modifications in peripheral blood mononuclear cells of patients treated with high-dose infusional doxorubicin. Blood.

[85] Liew CC, Ma J, Tang HC, Zheng R, Dempsey AA (2006). The peripheral blood transcriptome dynamically reflects system wide biology: a potential diagnostic tool. J Lab Clin Med.

[86] Todorova VK, Beggs ML, Delongchamp RR, Dhakal I, Makhoul I, Wei JY, Klimberg VS (2012). Transcriptome profiling of peripheral blood cells identifies potential biomarkers for doxorubicin cardiotoxicity in a rat model. PLoS One.

[87] Todorova VK, Makhoul I, Siegel ER, Wei J, Stone A, Carter W, Beggs ML, Owen A, Klimberg VS (2016). Biomarkers for Presymptomatic doxorubicin-induced cardiotoxicity in breast Cancer patients. PLoS One.

[88] Skitch A, Mital S, Mertens L, Liu P, Kantor P, Grosse-Wortmann L, Manlhiot C, Greenberg M, Nathan PC (2017). Novel approaches to the prediction, diagnosis and treatment of cardiac late effects in survivors of childhood cancer: a multi-Centre observational study. BMC Cancer.

[89] ISRCTN registry. The Cardiac CARE Trial - can heart muscle injury related to chemotherapy be prevented? 2017. [cited 2017 28 November]. Available from: https://www.isrctn.com/ISRCTN24439460?q=&filters=conditionCategory:Cancer&sort=&offset=2&totalResults=1958&page=1&pageSize=10&searchType=basic-search.

